# Insights into the diagnosis, vaccines, and control of *Taenia solium*, a zoonotic, neglected parasite

**DOI:** 10.1186/s13071-023-05989-6

**Published:** 2023-10-24

**Authors:** Md. Shahadat Hossain, Shafqat Shabir, Philip Toye, Lian F. Thomas, Franco H. Falcone

**Affiliations:** 1 Department of Parasitology, Bangladesh Agricultural University, Mymensingh, Bangladesh; 2 Institute of Parasitology, Justus Liebig University Giessen, Giessen, Germany; 3 International Livestock Research Institute, Nairobi, Kenya; 4 Institute of Infection, Veterinary and Ecological Sciences, University of Liverpool, Leahurst Campus, Neston, UK

**Keywords:** *Taenia solium*, Neurocysticercosis, Porcine cysticercosis, Taeniasis, Pig, Human, Vaccine, Diagnosis

## Abstract

*Taenia solium* taeniasis/cysticercosis (TSTC) is a foodborne, zoonotic neglected tropical disease affecting predominately low- and middle-income countries. Humans are definitive hosts for *T. solium*, whereas pigs act as intermediate hosts. Taeniasis, i.e. intestinal infection with adult *T. solium* in the human host, occurs through ingestion of undercooked pork infected with the larval stage (porcine cysticercosis, PCC). Human cysticercosis occurs after humans ingest *T. solium* eggs, acting as accidental intermediate hosts. Migration of cysticerci to the human brain results in neurocysticercosis (NCC), manifesting in a variety of clinical symptoms, most notably epilepsy. NCC is the leading cause of acquired epilepsy cases in endemic areas. PCC results in reduced pork value because of condemnation or the risk of condemnation of the meat. Available serological diagnostic tests for porcine and human cysticercosis are characterized by low sensitivity and are not cost-effective. An effective vaccine for *T. solium* cysticercosis in pigs has been developed, although it is not yet commercially available in all endemic countries, and still no vaccine is available for use in humans. This primer highlights the recent development in the field of diagnostic tests and vaccine production and explores possible strategies for future control and eradication of *T. solium*. In the absence of highly specific diagnostic tests and human vaccines, treatment of infected pigs and tapeworm carriers and prevention of disease transmission remain the principal means to interrupt the zoonotic cycle of *T. solium* in endemic countries.

**Graphical abstract:**

**Supplementary Information:**

The online version contains supplementary material available at 10.1186/s13071-023-05989-6.

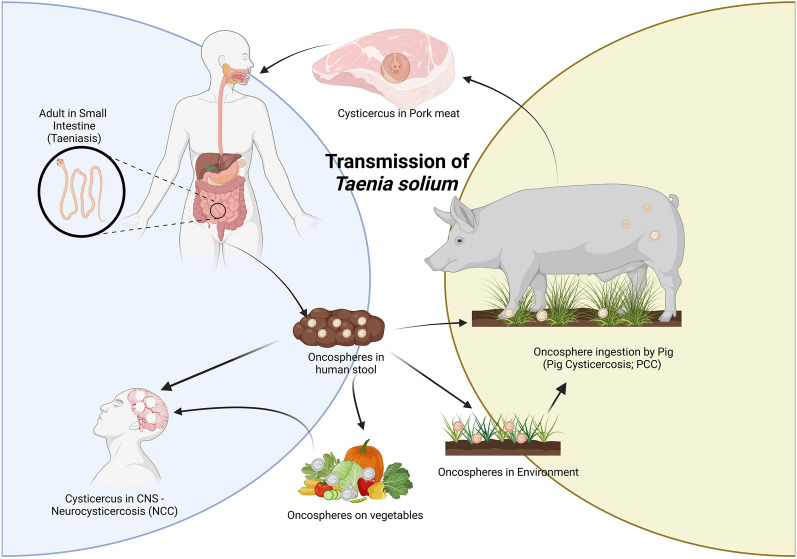

## Life cycle of *Taenia solium*

**Fig. 1:**
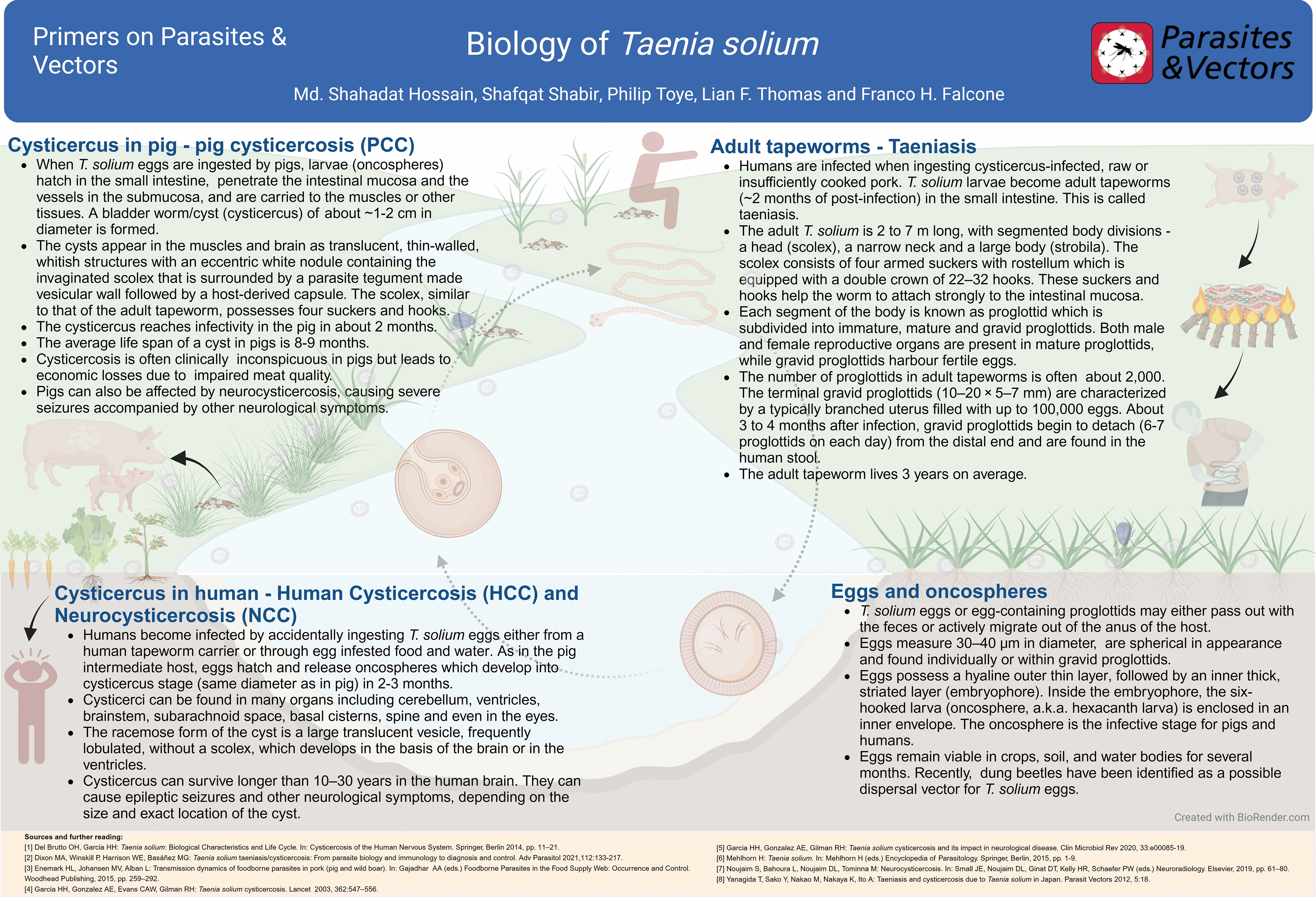
Biological characteristics and life cycle of *Taenia solium*. Humans harbor adult *T. solium* parasites in their small intestine. Adult parasites are hermaphrodites and release fertilised eggs or egg-containing mature proglottids, each containing up to 50,000–100,000 eggs, which are excreted with faecal material, contaminating the environment. Pigs are infected by oral ingestion of this faecal material or contaminated vegetation or feed. Upon ingestion, eggs hatch and oncospheres penetrate the gut wall, migrating to the musculature where they encyst, leading to pig cysticercosis (PCC). Human ingestion of undercooked or raw contaminated pork leads to the release of larval stages in the GI tract with subsequent development to an adult tapeworm (taeniasis). Accidental ingestion of mature eggs leads to human cysticercosis (HCC) and neurocysticercosis (NCC). Pigs do not harbor adult parasites; thus, infection is spread from humans to animals (anthropozoonosis). Created with BioRender.com.

The life cycle of *T. solium* involves humans as definitive hosts and pigs as intermediate hosts (Fig. 1) for a downloadable version of the poster see Additional File 1. Adult *T. solium* living in the human small intestine release eggs or egg-containing proglottids (50,000–100,000 eggs/proglottid) through stool into the environment. Several risk factors, like poor sanitation, open-air defecation, bio-fertilizer preparation from human stool and free-roaming pigs, help to transmit *T. solium* eggs in new areas and contaminate surface water as well as vegetation [1]. Pigs become infected by ingesting human stool or water/vegetation contaminated with *T. solium* eggs. Eggs hatch releasing the larval oncosphere in the pig intestine. Oncospheres penetrate the intestine wall, enter the bloodstream and develop into cysticerci. The larval cysticerci are mostly found in the striated muscles, subcutaneous tissues and central nervous system (CNS), resulting in porcine cysticercosis (PCC). Pigs with larval cysticerci in the brain suffer from neurocysticercosis (NCC) and can develop clinical signs and suffer from seizures, while some pigs may have some autonomic signs, like chewing motions with foamy salivation and ear stiffening [2]. Human taeniasis results from ingestion of raw or undercooked pork infected with viable *T. solium* cysticerci [3]. In the human intestine, adult tapeworms may produce some non-specific symptoms, such as abdominal pain, nausea, diarrhea, or constipation, approximately 8 weeks after infection [4, 5]. Tapeworm carriers shed *T. solium* eggs and proglottids through stool that can again contaminate the environment. Additionally, humans may be infected by ingestion of *T. solium* eggs with water, vegetables, or raw salad items contaminated with viable eggs from a tapeworm carrier stool. In this case, eggs develop into cysts and lodge within tissues such as the CNS, skeletal muscles, or subcutaneous tissues, resulting in human cysticercosis (HCC). When larval cysts develop within in the CNS, they cause human NCC [6]. NCC can result in a wide range of neurological and psychiatric manifestations including seizures/epilepsy, severe headaches, focal deficits and signs of increased intracranial pressure [7].

## *Taenia solium* tapeworm, a poverty-related neglected tropical disease (NTD)

*Taenia solium* cysticercosis is one of the 20 major neglected tropical diseases and a member of the neglected zoonotic disease (NZD) subset listed as a potentially eradicable disease by the World Health Organization (WHO), while the World Organization for Animal Health (WOAH) identifies PCC as a notifiable disease. *Taenia solium* infection in both human and pig is endemic in low- and middle-income countries (LMIC) of Latin America, sub-Saharan Africa, South and Southeast Asia [8]. Imported cases of human cysticercosis have been reported in most Western European countries[9], though recent studies have reported autochthonous cases of NCC in Europe, with eastern European countries most at risk [10–13]. A meta-analysis of sero-epidemiological data from endemic areas reported 7.30% (95% CI [4.23–12.31]), 4.08% (95% CI [2.77–5.95]) and 3.98% (95% CI [2.81–5.61]) prevalence of circulating *T*. *solium* antigens, for Africa, Latin America and Asia, respectively. Seroprevalence estimates of *T*. *solium* antibodies were 17.37% (95% CI [3.33–56.20]), 13.03% (95% CI [9.95–16.88]) and 15.68% (95% CI [10.25–23.24]), respectively [14]. NCCs affects between 2.5 and 8.3 million people annually with a disability-adjusted life year (DALY) burden of 2.8 million (95% CI) [7]. The most common clinical manifestations associated with NCC are seizures/epilepsy, followed by headaches, focal deficits and signs of increased intracranial pressure [15]. It is one of the leading cause of acquired epilepsy (30% of epilepsy cases) in endemic countries [16]. The reported prevalence of NCC in people with epilepsy in endemic areas of sub-Saharan Africa was 22% (95% CI, 17.0–27.0) [17]. The estimated number of individuals with epilepsy due to NCC are 0.45–1.35 million in Latin America, 1 million in India and 0.31–4.6 million in Africa [18]. Infection of pigs with the larval stage of *T. solium* results in PCC. It is widely prevalent in sub-Saharan Africa with a meta-analysis reporting a high-pooled prevalence using available diagnostic tests (17%, 95% CI: 14–20%) across the continent [19] but hyper-endemic (i.e. persistent, high levels of disease occurrence) in Central Africa [8, 20]. In this hyper-endemic region, the recent reported prevalence of PCC was 45.6% (95% CI, 40.2–51.0) by antigen-ELISA, 24.8% (95% CI, 20.1–30.5) by antibody-ELISA, 15.5% (95% CI, 12.3–18.7) and 9.2% (95% CI, 7.9–10.7) by tongue palpation in the Democratic Republic of the Congo (DRC), Cameroon, Burundi and Rwanda, respectively [21–24]. Taeniasis has been poorly studied and is underreported in many endemic countries. The reported prevalence of taeniasis varies between 0% (95% CI [0.00–1.62]) and 17.25% (95% CI [14.55–20.23]) using different diagnostic techniques in Africa, Latin America and Asia [14]. Even low prevalence of taeniasis can sustain high prevalence of PCC and NCC because of the high number of eggs shed and the ability of a single taeniasis case to infect many pigs and humans.

While taeniasis itself has little impact on human health despite rare sequelae, cysticercosis not only has direct effects on human and animal health but also on the socioeconomic status of the former. NCC-associated epilepsy has direct effects on human health-related expenses like diagnosis, treatment and medicine cost for patients during hospitalization. Besides, indirect effects of NCC are linked to people who either become unemployed or are unable to work because of epilepsy [25]. PCC results in carcass condemnation and decreased value of pigs, which is estimated to cause 20–60% production losses in pig-raising countries or pig-based industries [25–28]. A regional study in western and central African countries estimated annual losses of about 25 million euros [20]. In many countries pig farming is an important source of emergency cash flow for marginalized households. In Uganda, for example, pig farming solely supports the livelihoods of > 1.1 million households [29].

**Fig. 2:**
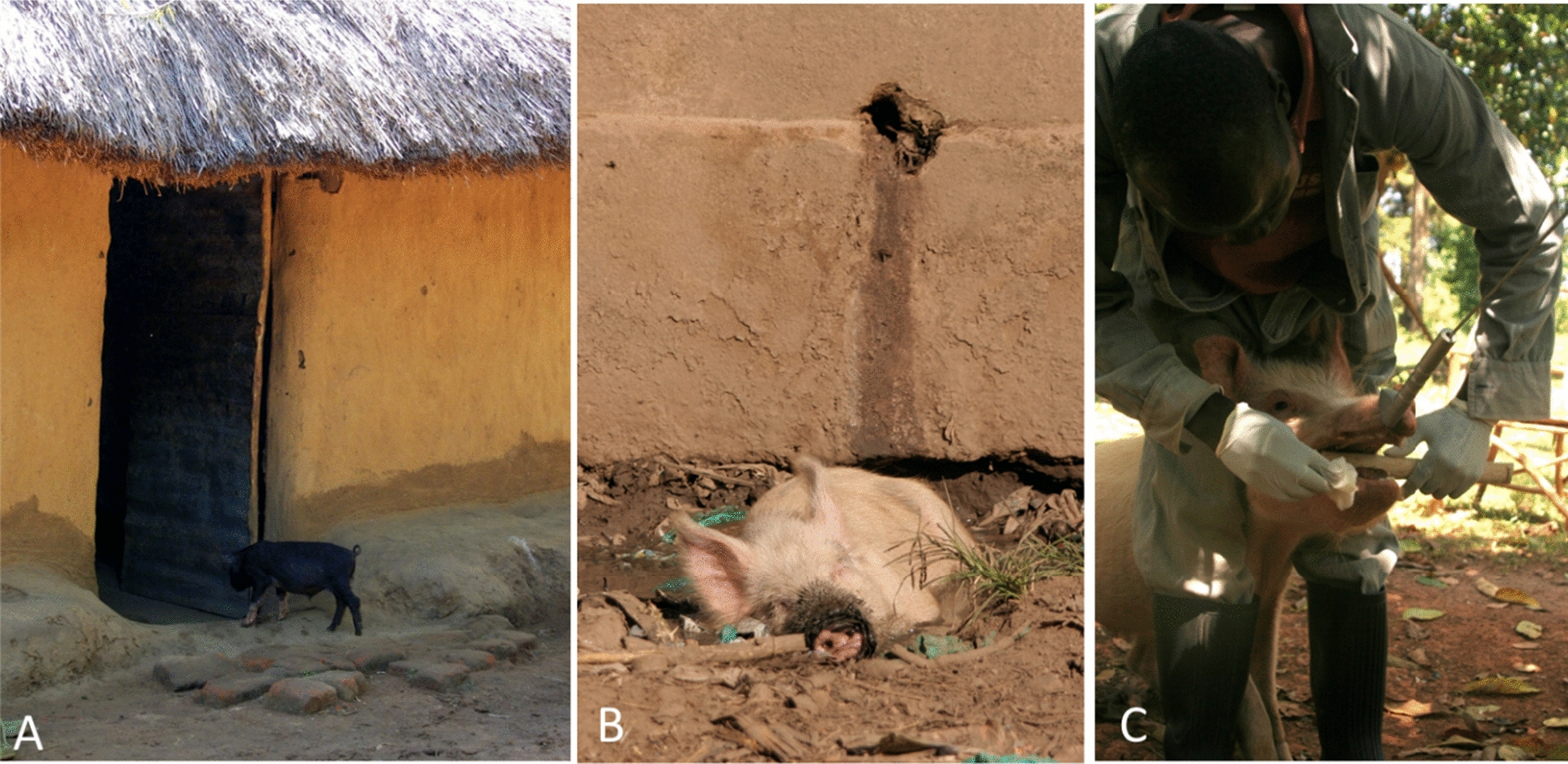
**A** Lack of separation between pig and human habitats, as illustrated in (**A**); where pigs are allowed to roam freely, the potential for pigs to come into contact with human faecal material containing infective eggs is increased. **B** Pig wallowing in a pit containing human excrement from a draining latrine. **C** Assessment of pig cysticercosis by tongue palpation. All photographs courtesy of Lian F. Thomas

TSTC is a focal disease, affecting the poorest communities of developing countries. In many of these communities pigs are raised in free-roaming systems (Fig. 2), and low latrine coverage results in open defecation, exposing roaming pigs to stool of tapeworm carriers [14, 29]. Home slaughter of those pigs, without appropriate veterinary inspection, is another practice among smallholder farmers, which increases the zoonotic risk in the community [30]. In this way, *T. solium* transmission persists, ultimately increasing the global burden of this NTD and affecting the livelihood of the pig-raising community in endemic regions.

## *Taenia solium*: three recent advances

### Rapid diagnosis of *T. solium* infection

As cysticercosis is mainly a problem in LMICs, diagnostic technologies not requiring specialized equipment or highly trained personnel would be advantageous. This has prompted the development of rapid detection technologies for *T. solium* infection, which has shown promising capability in recent times using recombinant antigens [31].

A lateral flow assay-based diagnostic tool was developed using up-converting phosphor (UCP) nanoparticles to detect binding of UCP to TSOL18 (oncosphere-stage protein) and GP50 (cystic larval-stage protein) antigen of *T. solium*, whose signal is then transferred to a biosensor for analysis. This technology has higher sensitivity and specificity compared with ELISA, with a sensitivity of 93.59% and 97.44% for TSOL18 and GP50, respectively, and a specificity of 100% for both antigens [32]. However, it needs to be stressed that UCP technology is not equipment-independent, as the technology is based on conversion of infrared to visible light.

Another study developed a point-of-care (POC) assay using quantum dot nanoparticles in a lateral flow assay format and detected rT24h antigen of *T. solium* in NCC patients with a mobile phone reader. The assay specificity was 99% (95–100%) while sensitivity was 89% (79–95%) in patients with two or more viable cysts [33].

Multiplex Bead Assay is one of the recombinant antigen-based diagnostic tools, which can estimate the intensity of the antibody response and allow direct comparison of antibody levels between samples. In one study, this assay was used for evaluating two recombinant antigens, rT24H (*T. solium*-specific) and r2B2t (*Echinococcus granulosus*-specific) for simultaneous and differential diagnosis of NCC and cystic echinococcosis. For the diagnosis of NCC, the sensitivity and specificity of this assay ranged from 57.94 to 63.49% and 90.87–91.30%, respectively [34].

### Molecular identification of *T. solium*

DNA-based diagnostic methods have been developed to identify *T. solium* DNA specifically, either from human stool or directly from tapeworm-derived segments. LAMP (loop-mediated isothermal amplification) is a DNA-based isothermal PCR technique, which has emerged as a good diagnostic tool in the last decade for the detection of different taeniids at species level. A multiplex LAMP (mLAMP) assay uses mitochondrial cytochrome oxidase subunit 1 (COX1) marker in combination with dot enzyme-linked immunosorbent assay (dot-ELISA). This assay was successfully used to identify *Taenia* species from genomic and copro-DNA samples with 100% specificity, making it the first field-based test for sensitive and specific identification of human *Taenia* species [26]. Lyophilized LAMP has been developed with long shelf life and does not require any type of cold chain support, which can therefore facilitate its use for diagnosing *T. solium* in endemic countries [35, 36]. Recently, quantitative molecular approaches like quantitative polymerase chain reaction (qPCR) have become an attractive platform for biomarker and diagnostic test development for identifying specific *T. solium* sequences. In the *T. solium* genome, TsolR13 sequence has shown high sensitivity (97.3%) and specificity (100%) for diagnosis of NCC [37]. Another study used qPCR for the differentiation among *Taenia solium*, *T. saginata* and *T. asiatica* in human stool. This study targeted the internal transcribed spacer I (ITS-1) gene of *Taenia solium* and COX1 gene of *T. saginata* and *T. asiatica*. For the diagnosis of taeniasis, this study showed 94% sensitivity and 98% specificity [38].

### Recombinant vaccine development for pig immunization

Vaccination of pigs is an efficient method to reduce human exposure to infected pork, which ultimately reduces both taeniasis and cysticercosis. For pig vaccine development, different *T. solium* antigens have been explored for their suitability, for instance, *T. solium* scolex protein antigen [39]; TSOL16, TSOL18, TSOL45-1A, TSOL45-1B (oncosphere protein) [40, 41]; *T. solium* paramyosin, a muscle and tegument protein [42]. Among those antigens, TSOL18 has been identified as the most promising candidate for recombinant vaccine development against PCC. This vaccine can provide 99.5% protection against PCC [43]. TSOL18 vaccine was first commercialised by GALVmed, Indian Immunologics (IIL) and University of Melbourne [44]. The vaccine was then licensed in 2016 in India. Field trials in Cameroon [45] and Nepal [46] evaluated a combination of TSOL18 vaccine (Cysvax™) and Oxfendazole (Paranthic 10%™) drug against PCC. Oxfendazole removes existing cyst infection in pig while TSOL18 checks subsequent infection with *T. solium*. This combined therapy increases the protection level up to 99.7% of all animals exposed to subsequent infection and results in significant reduction in prevalence of PCC in endemic countries [47–50]. Another two synthetic vaccine candidates against *T. solium* SP3VAC (KETc7, KETc1 and KETc12) and a modified parenterally administered SP3Vac-phage version have undergone trials in Mexico [43]. Whilst TSOL18 vaccine (Cysvax™) is now commercially available at a relatively low cost (US$ 2.31) in many of the endemic countries, there is evidence from Uganda that pig farmers are not yet willing to pay for the vaccine without a market premium for vaccinated pigs and in the absence of intrinsic health benefits for the vaccinated animals [28].

## *Taenia solium*: three areas ripe for research

### Developing human vaccines

Most vaccine research work on *T. solium* to date has primarily focused on identifying candidate vaccines for pig to control PCC in endemic countries. Animal vaccines are comparatively easier and cheaper to develop than human vaccines. Therefore, until now human vaccination has not been considered an effective control strategy for TSTC in humans [47]. *Taenia solium* calreticulin, a tegument protein of the parasite, is essential for embryogenesis, oogenesis and spermiogenesis. This protein has been explored as a candidate vaccine target, and several studies showed reduced worm burden in an experimental hamster model of taeniasis [51–53]. In another study, fatty acid-binding proteins of *T. solium* showed only 45% reduction of parasite load against an intraperitoneal challenge with *T. crassiceps* cysts in a murine model of cysticercosis [54]. More candidate antigens of *T. solium* still need to be explored to identify a reliable and affordable vaccine for human use. While there is currently little willingness to pay by farmers for the highly effective TSOL18 vaccine for pig, the development and incorporation of a human vaccine into childhood vaccination schedules may be an option to significantly reduce infection in exposed populations.

### Further improving available diagnostic tests

The available diagnostic tests are still not fully suitable for sensitive and specific field level diagnosis of TSTC. To help overcome this gap, WHO defined four new target product profiles (TPPs) for researchers, diagnostic developers and manufacturers to develop effective and useful diagnostic tests for *T. solium*. Among four TPPs, two were classified for taeniasis-specific test and POC test development, one for NCC POC test development and one for porcine cysticercosis-specific diagnostic test development [31]. TPPs mandate that for a specific test of taeniasis, the sensitivity and specificity should be ≥ 95% and 99%, respectively. Furthermore, the cost of the test should be between 0.5 and 2 USD per test and the developed test kit should be stable for 24–36 months at 2–40 °C. For the POC test of taeniasis, sensitivity should range between 95 and 99% and specificity between 80 and 99% with same cost and storage conditions as mentioned above. For POC test for NCC the sensitivity should be between 98–99% with 90–95% specificity and the cost of the test should be between 2–3 USD along with 24–36-month stability at 2–30 °C. However, TPPs for PCC diagnostic test need 50–70% sensitivity for around 50 cysts and 80–90% for > 50 cysts, and the test should at least detect one single viable cyst in pig, while the specificity should range between 95 and 98%. The test should not show cross-reaction with either *Taenia* species or other parasites and not show any positive response in the absence of viable cysts. In addition, the cost of this test should be 0.5–2 USD per test with 24–36 month stability at 2–40 °C temperature [31].

The current reference methods for PCC and NCC are the full carcass muscle/brain dissection and imaging techniques, which are not feasible economically in developing countries. There is no dry LAMP format for the detection of *T. solium* infections in both humans and animals. Moreover, specific methods and diagnostic tools are still unavailable for inspecting vegetables or other possible transmission vehicles (soil, water) for *T. solium* eggs [55].

### Supporting intervention design and evaluation: transmission modelling, economic evaluation and risk mapping

To tackle *T. solium* taeniasis and cysticercosis in endemic countries, the WHO aims to scale up the intervention methods used to control *T. solium*. WHO projected that 27% of endemic countries will achieve intensified *T. solium* control in hyperendemic areas by 2030 [56]. This target is achievable by revealing unknown transmission dynamics of *T. solium*, including risk factor identification in both host and environment. In endemic countries, spatial distribution and risk factor analysis for human and porcine cysticercosis is one of the best options to unravel the transmission patterns of *T. solium*. A new agent-based model, CystiAgent, has created a model for analysing spatial and behavioural features of *T. solium* transmission in northern Peru. This model has the ability to represent key spatial and environmental features of transmission and simulate spatially targeted interventions, such as ring strategy [57]. Another model, CystiHuman, based on CystiAgent, was also developed to simulate human NCC and associated pathologies in the endemic community setting of Peru [58]. A deterministic, compartmental transmission model for pig and human transmission of *T. solium* (EPICYST) has also been developed [59]. These models can support the design and *ex ante* assessment of control options for *T. solium* but maximising the utility of these models will require continued collaborative efforts between modelling teams to improve and harmonise models and model parameters [59, 60]. As well as determining the efficacy of control programmes in reducing infection pressure, it is important to quantify the impact on burden of disease. Whilst human health burden can be measured in DALYs, for the evaluation of zoonotic disease control strategies such as for *T. solium*, a novel metric, zoonotic disability-adjusted life years (zDALY), may be more appropriate [61]. zDALYs estimate the actual impact of a zoonotic disease across both animal and human health and may be used to better assess the cost-effectiveness of a control programme across multiple sectors [62]. Appropriate geographical targeting of control programmes requires accurate endemicity mapping. Recently, WHO updated a new endemicity map at country level in 2022, but lack of subnational mapping is still a major challenge to achieving intensified *T. solium* control milestones [13]. A recent study in Uganda mapped all the available *T. solium* prevalence data and the PCC risk factors to obtain a complete insight into the *T. solium* landscape and subnational variation of indicators [63]. Better understanding of adult *T. solium* biology with identification of environmental dynamics of eggs and risk factors analysis of pig-to-people and environment-to-pig transmission will be paramount to achieving WHO 2030 targets.

## Conclusions

Taken together, although eradication of TSTC appears possible in principle, it is still far from being achieved. Some very effective components, such as combined treatment/vaccination with oxfendazole and TSOL18, are available, but there are still economic and sociocultural factors hindering their implementation in many endemic countries, and affordable, effective diagnostic technologies still lag behind. In the meantime, an achievable, immediate objective will be to implement highly targeted, small-scale control programmes in the most impacted areas by taking advantage of the improved new mapping tools combined with better transmission models.

### Supplementary Information

**Additional file 1:** Biological characteristics and life cycle of *Taenia solium*. Humans harbor adult *T. solium* parasites in their small intestine. Adult parasites are hermaphrodites and release fertilised eggs or egg-containing mature proglottids, each containing up to 50,000–100,000 eggs, which are excreted with faecal material, contaminating the environment. Pigs are infected by oral ingestion of this faecal material or contaminated vegetation or feed. Upon ingestion, eggs hatch and oncospheres penetrate the gut wall, migrating to the musculature where they encyst, leading to pig cysticercosis (PCC). Human ingestion of undercooked or raw contaminated pork leads to the release of larval stages in the GI tract with subsequent development to an adult tapeworm (taeniasis). Accidental ingestion of mature eggs leads to human cysticercosis (HCC) and neurocysticercosis (NCC). Pigs do not harbor adult parasites; thus, infection is spread from humans to animals (anthropozoonosis). Created with BioRender.com.

## Data Availability

Not applicable.

## Abbreviations

CNS: Central nervous system
DALY: Disability-adjusted life years
HCC: Human cysticercosis
LAMP: Loop-mediated isothermal amplification
LMIC: Low and middle income country
NTD: Neglected tropical disease
NCC: Neurocysticercosis
NZD: Neglected zoonotic disease
PCC: Porcine cysticercosis
POC: Point of care
PWE: People with epilepsy
TPPs: Target product profiles
TSTC: *Taenia solium* taeniasis/cysticercosis
WHO: World Health Organization
WOAH: World Organization for Animal Health
zDALY: Zoonotic disability-adjusted life years
