# Supplementary figures and images for: Insights into the diagnosis, vaccines, and control of Taenia solium, a zoonotic, neglected parasite
Source: Parasit Vectors. 2023 Oct 24;16:380. doi: 10.1186/s13071-023-05989-6 (PMC10594694; doi:10.1186/s13071-023-05989-6)

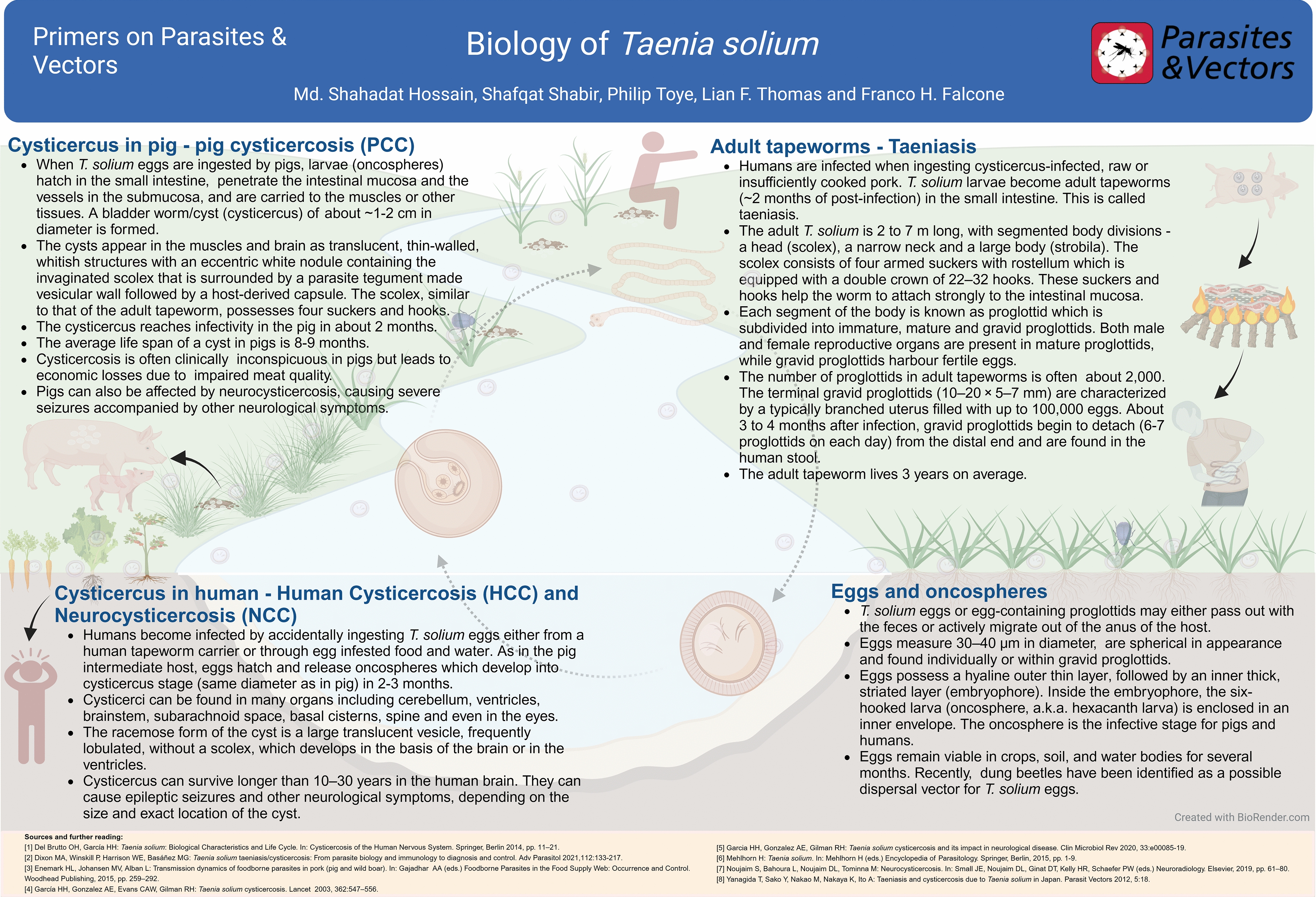

Supplement: Supplementary file 1 — Additional file 1: Biological characteristics and life cycle of Taenia solium. Humans harbor adult T. solium parasites in their small intestine. Adult parasites are hermaphrodites and release fertilised eggs or egg-containing mature proglottids, each containing up to 50,000–100,000 eggs, which are excreted with faecal material, contaminating the environment. Pigs are infected by oral ingestion of this faecal material or contaminated vegetation or feed. Upon ingestion, eggs hatch and oncospheres penetrate the gut wall, migrating to the musculature where they encyst, leading to pig cysticercosis (PCC). Human ingestion of undercooked or raw contaminated pork leads to the release of larval stages in the GI tract with subsequent development to an adult tapeworm (taeniasis). Accidental ingestion of mature eggs leads to human cysticercosis (HCC) and neurocysticercosis (NCC). Pigs do not harbor adult parasites; thus, infection is spread from humans to animals (anthropozoonosis). Created with BioRender.com. [file 13071_2023_5989_MOESM1_ESM.jpg]
